# Application of Empirical Mode Decomposition for Decoding Perception of Faces Using Magnetoencephalography

**DOI:** 10.3390/s21186235

**Published:** 2021-09-17

**Authors:** Chun-Hsien Hsu, Ya-Ning Wu

**Affiliations:** Institute of Cognitive Neuroscience, National Central University, Taoyuan City 320317, Taiwan; neurolang@g.ncu.edu.tw

**Keywords:** magnetoencephalography (MEG), empirical mode decomposition (EMD), neural decoding, face perception

## Abstract

Neural decoding is useful to explore the timing and source location in which the brain encodes information. Higher classification accuracy means that an analysis is more likely to succeed in extracting useful information from noises. In this paper, we present the application of a nonlinear, nonstationary signal decomposition technique—the empirical mode decomposition (EMD), on MEG data. We discuss the fundamental concepts and importance of nonlinear methods when it comes to analyzing brainwave signals and demonstrate the procedure on a set of open-source MEG facial recognition task dataset. The improved clarity of data allowed further decoding analysis to capture distinguishing features between conditions that were formerly over-looked in the existing literature, while raising interesting questions concerning hemispheric dominance to the encoding process of facial and identity information.

## 1. Introduction

The human brain is a complex system that generates nonstationary and nonlinear oscillatory activities. When processing brainwave signals for feature analysis, however, linear approximation techniques such as Fast Fourier Transform (FFT) and wavelet transform (WT) have been extensively employed as the norm for clinical and research purposes despite their limitations in handling signals with statistical properties that change along the time domain [1]. Although these methods may be sufficient for many practical purposes, they are incapable of properly estimating a nonlinear function on a long time-scale, which results in inaccurate representation of the time-frequency fluctuation of brainwave signals and information loss about phase and amplitudes [2]. Empirical mode decomposition (EMD) is a nonlinear transforming method that decomposes times series into multiple components, namely the intrinsic mode functions (IMFs). IMFs are acquired by repeating the sifting process, which connects local extrema into envelopes and subtracts the moving average repeatedly [2]. This iterative technique makes use of the time series of signals and could perform without any requirement for priori assumptions, differentiating itself from the traditional and commonly used linear methods such as FFT, wavelet transform and Matching Pursuit (MP). Masking EMD builds its foundation on empirical mode decomposition (EMD), a method proposed by Huang et al. in 1998 [3]. EMD differs from EMD specifically in that it addresses the mode mixing problem and thus improved the stability of decomposition concerning noises [2]. To sum up, as a local method, EMD is based on the data’s own structure, it is able to preserve the nonstationary and nonlinear characteristics after decomposition [4]. In this study, we performed neural decoding and permutation test analysis on a set of magnetoencephalography (MEG) data processed by EMD. Despite HHT’s potential in increasing sensitivity and accuracy to high nonlinear complexity of brain wave signals, few studies explored the combinatory use of EMD and neural decoding in depth [2]. Our goal was to take the nonlinear characteristics of brainwaves into consideration when performing neural analysis. Compared to past analysis procedures using the traditional linear techniques, we aimed to inspect this method’s sensitivity to neural activities and ability to identify pattern differences between conditions. If EMD was capable of capturing nonstationary features that may not be easily detectable by the commonly used linear methods, it could tremendously improve and benefit clinical assessments and neural activity analysis. For the analysis material, we used a set of open-source neural recordings collected by Wakeman and Henson [5]. This is a publicly available dataset that has been utilized frequently in recent years, often for testing newly developed classification models and algorithms [6,7,8,9]. The dataset consists of facial recognition task data, in which sixteen participants were exposed to a series of famous, unfamiliar and scrambled faces. More details on the task procedure would be provided in the method section.

One thing we observed after searching through past research studies is a shared difficulty or less accurate performance in detecting activity pattern differences between the familiar and unfamiliar face stimuli conditions. In Wakeman and Henson’s original study, for instance, significant clusters did not survive testing correction [5]. Follow-up studies or algorithm testing done by other researchers also either showed similar low decoding accuracies between familiar and unfamiliar stimuli conditions [8], or simply discard one set/combine both sets of the facial stimuli into one (familiar and unfamiliar) [10,11,12]. In spite of the intuition that familiar faces should provide more information than unfamiliar faces, the results from these studies seemed to portray another story. We were interested in the question of whether further information could be extracted when a nonlinear method is employed instead of the linear ones. With our ultimate goal aiming at providing new perspectives on ways in conducting neural decoding procedures, we put EMD analysis on MEG data to test. We utilized EMD for signal decomposition and investigated the involvement of specific frequency ranges of brain oscillation and focal regions of interests. Additionally, we utilized simulation models to verify the precision of our source reconstruction results. The dilemma of the inverse problem of source reconstruction is something that has not been addressed properly in the current development of nonlinear method applications in neural data processing. Considering the relatively new stage of exploration on nonlinear methods, the source reconstruction approach that is specifically customized to nonlinear input is rare, if any, as far as the authors are concerned. Therefore, to address the long unresolved question in the field that the association between the IMFs and nodes on the cortex may be unclear after time series decomposition, we performed a set of basic simulations and modeled three simultaneously active cortical sources.

## 2. Materials and Methods

### 2.1. Dataset and Experimental Design

#### 2.1.1. MEG Dataset of 16 Participants and Experimental Design

The data of 16 participants performing a simple perceptual task are freely available on the OpenNeuro platform [5]. The accession number is ds000117. There were 19 participants in the age range of 23 to 37 according to Wakeman and Henson’s original work [5]. All were Caucasian except for one Asian participant [5]. However, only 16 participants’ data are currently available on the open-source platform, which were from 7 females and 9 males. Since this experiment has been discussed in detail elsewhere [5], only a brief introduction is provided in this article. In the experiment, participants were instructed to make judgments on how symmetric they perceived a face stimulus to be, by pressing one of the two keys available to indicate whether they thought it was more or less symmetrical than an average face. The stimuli set consisted of grayscale photographs of famous, unfamiliar, and scrambled faces. A fixation cross appeared on screen for a duration between 400 and 600 ms at random. The target stimulus of either a face or scrambled face was then presented to the participants for a duration between 800 and 1000 ms. The original dataset comprises MEG and EEG simultaneous recordings using Elekta Neuromag Vectorview 306 system. We focused on the MEG portion in our analysis.

#### 2.1.2. Simulation

We performed a set of simulations by modeling three simultaneously active cortical sources. To conduct the simulation, a boundary element method (BEM) volume conduction Model was created using T1 images of the participant 2 from Wakeman and Henson’s. We simulated evoked responses generated at three anatomical labels from the aparc.a2009s. Parcellation [13], including two evoked delta-band (3 Hz) responses at the left and the right Supramarginal gyrus and one theta-band source (7 Hz) in the right inferior frontal gyrus. We chose these two bands as they were the primary focus in our study.

### 2.2. Data Preprocessing

**Figure 1:**
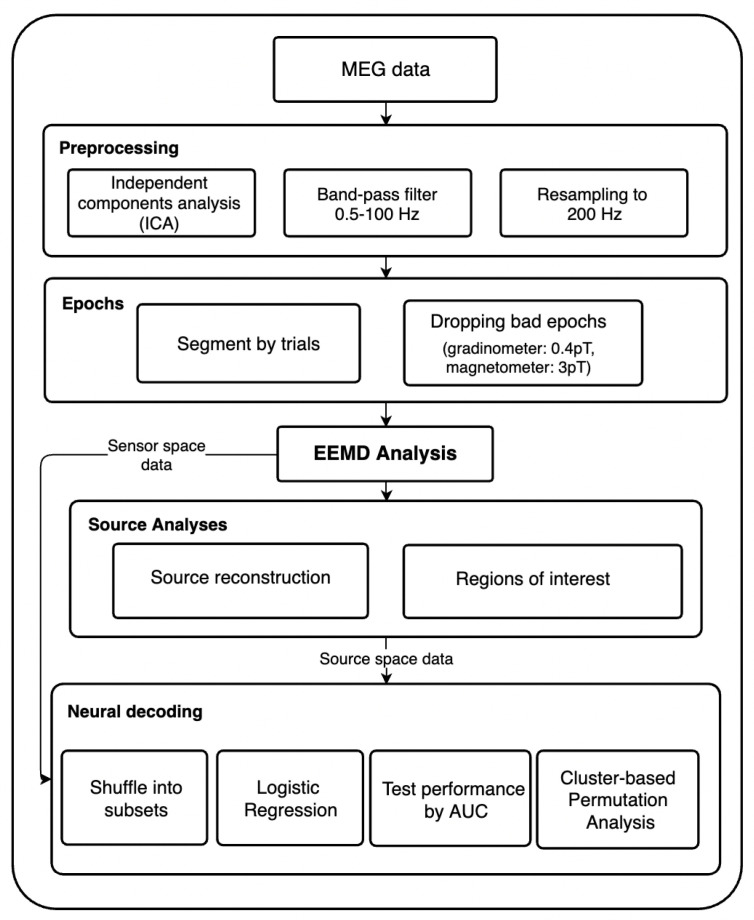
This figure shows the step-by-step data processing procedures along with all the analyses that we performed.

Independent Components Analysis (ICA) was employed in order to reduce signal contamination from noises such as heart beats and eye blinks. By using ICA on continuous data, the signal was able to be separated into additive non-Gaussian subcomponents of the same length of the original recording. Each of these ICA components were statistically independent from one another. Artifacts such as eye movement and heart beats are notably characterized by steady patterns that recur throughout the whole recording. Components as such were identified as noises and removed manually. A band-pass filter from 0.5 to 100 Hz was then applied. Afterwards, the MEG data were resampled from 1000 Hz to 200 Hz in an attempt to reduce the data size and computational demands. For better visualization, the procedure is shown in Figure 1.

The data were then segmented into epochs. Each epoch was 1100 ms long, starting at 100 ms pre-stimulus onset and ending 1000 s after the onset. There were three conditions in this study (famous face, unfamiliar face, and scrambled face) that were eventually categorized. Using the recordings from 100 ms pre-stimulus to onset (0 ms) as the baseline, artifact rejection was performed on the epochs in an attempt to reduce biological and environmental interferences. Corrupted data with peak-to-peak amplitude signals exceeding a predetermined rejection parameter were removed.

### 2.3. EMD Analysis

The masking EMD analysis [4] was performed on each participants’ epochs data, and each epoch was then decomposed into eight IMF components. Since the last component (IMF 8) decomposed from the original epoch signal was the residual trend, it was removed before performing further analysis. The dominant frequencies of the remaining IMFs (1-7) as defined by peak amplitude in Hilbert spectra were approximately distributed in the range of certain brainwave oscillations.

### 2.4. Neural Decoding

The following decoding process was performed by using Scikit-Learn and MNE-python on the original epoch data and IMF 4 to 6 components of each condition [14,15]. For each participant, the data for each stimuli condition were shuffled and divided into 30 subsets. Next, three pairs of conditions (famous and unfamiliar faces, famous and scrambled faces, unfamiliar and scrambled faces) were formed to make further comparisons. Every subset would be used once as a testing set as the others were used as the training sets using the logistic regression model to test whether the MEG activities could predict labels of stimuli condition. The performance of classification was evaluated by calculating the area under the curve (AUC) of the receiver characteristic operator that would fall in the range of 0–1.0. If the AUC score is above 0.5, then the prediction accuracy is above chance level. To evaluate the time course of the emergence of facial representation, classification analyses were performed for each time frame after stimulus onsets. Furthermore, the cluster-based permutation analyses (1000 times of permutation) were applied to evaluate the significance of classification models.

### 2.5. MEG Source Reconstruction

Participants’ structural MR images were processed in FreeSurfer (Laboratory for Computational Neuroimaging at the Athinoula A. Martinos Center for Biomedical Imaging, Charlestown, MA, USA) to create a cortical reconstruction of each participant’s brain [16]. MNE-python was then used to calculate a cortically constrained L2 minimum-norm solution for each participant’s MEG data with 5124 sources on participant’s cortical surface. The boundary-element model method was used to compute the forward solution, which estimates the resulting magnetic field at each MEG sensor by the activity at each of the 5124 sources. Next, the inverse solution was employed to identify the spatiotemporal distribution of activity over sources that best account for each participant’s 138 averaged MEG data. The resulting minimum-norm estimates were converted into a dynamic statistical parameter map (dSPM), which measured the noise-normalized activation at each source to avoid inaccuracies of standard minimum-norm calculations [17]. The noise-covariance matrix was estimated from the empty-room data, while each participant’s cortical surface was normalized onto a standard brain supported by Freesurfer.

### 2.6. Region of Interest (ROI)

To elucidate the neural correlates supporting the emergence of facial representation, we performed the decoding analyses using single-trial dSPM activities in regions of interest (ROI): fusiform, inferiortemporal, entorhinal, frontal, medial orbital frontal cortex, and temporal pole. Data from the left and right hemispheres were analyzed and modeled to compare their intensity and timings of activities. After performing the above steps on the original and decomposed data of each participant, we average the outputs of all participants for each condition and ROI.

## 3. Results

### 3.1. Hilbert Spectra

**Figure 2:**
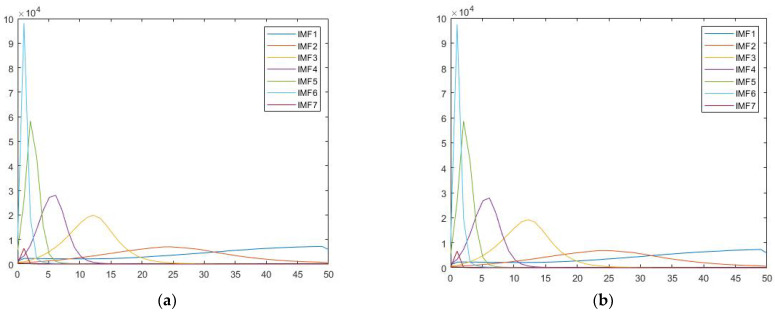
This figure shows the Hilbert spectra for (**a**) famous, (**b**) scrambled face conditions for participant 001 in the dataset. The *x*-axis is frequency (Hz), and the *y*-axis is energy (a.u.).

We obtained IMF through the signal decomposition process of EMD and Hilbert spectral analysis. Similar to results of a previous study [5], while individual differences could be observed in their energy levels, the corresponding frequency range of these peak values were stably similar among participants. It is worthwhile to mention that the frequency distribution for each IMF corresponds to different physiological rhythms from the brain. For instance, IMF 1 contains high frequency waves, while IMF2 and IMF3 were mainly in the range of alpha and beta waves, respectively. The dominant frequency of averaged IMF4 across participants fell in the range of theta wave oscillation (5.87 Hz), while those of IMF5 and IMF6 (IMF5 = 2.04 Hz, IMF6 = 1.02 Hz) were in the range of delta wave oscillation. The following graph is an example showing the IMF frequency distribution spectra of the famous and scrambled condition for a single participant (Figure 2). Since our pilot did not show any significant activity information on IMFs 1-3, this study focused on analysis on IMFs 4-6.

### 3.2. dSPM

**Figure 3:**
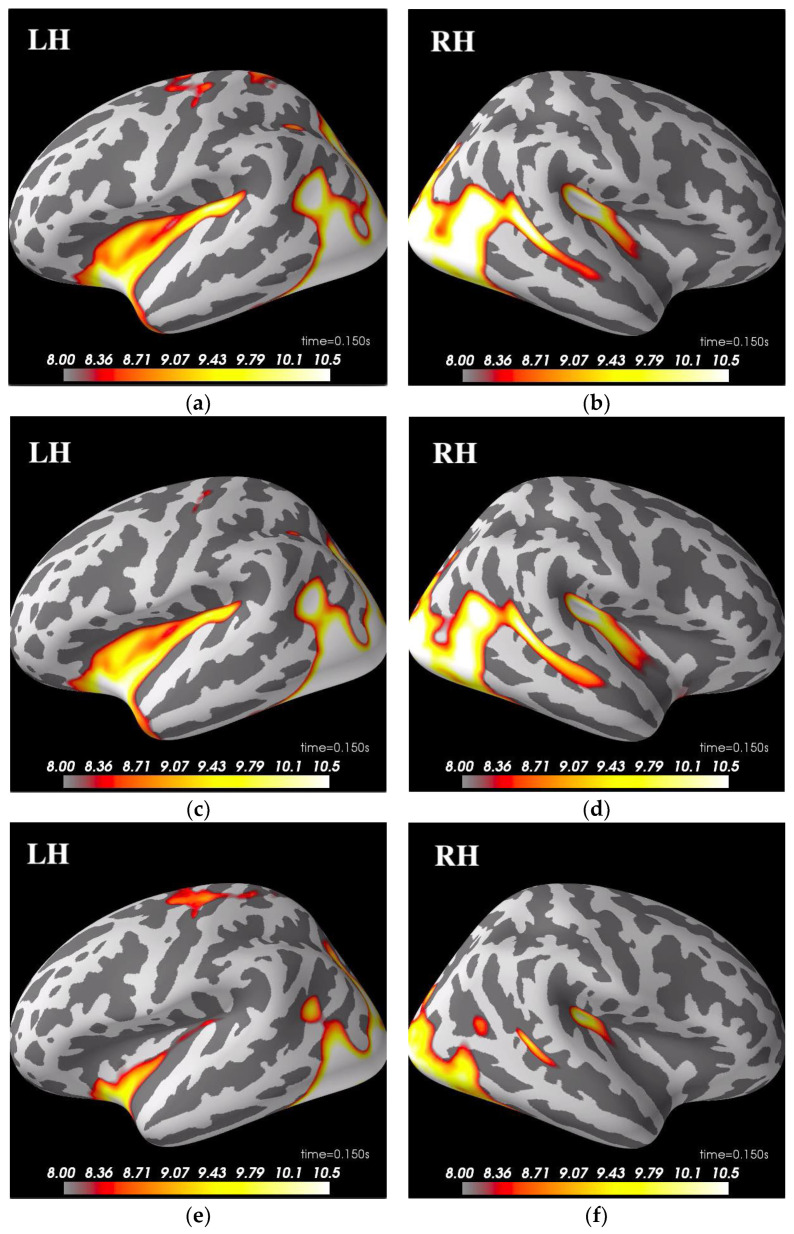

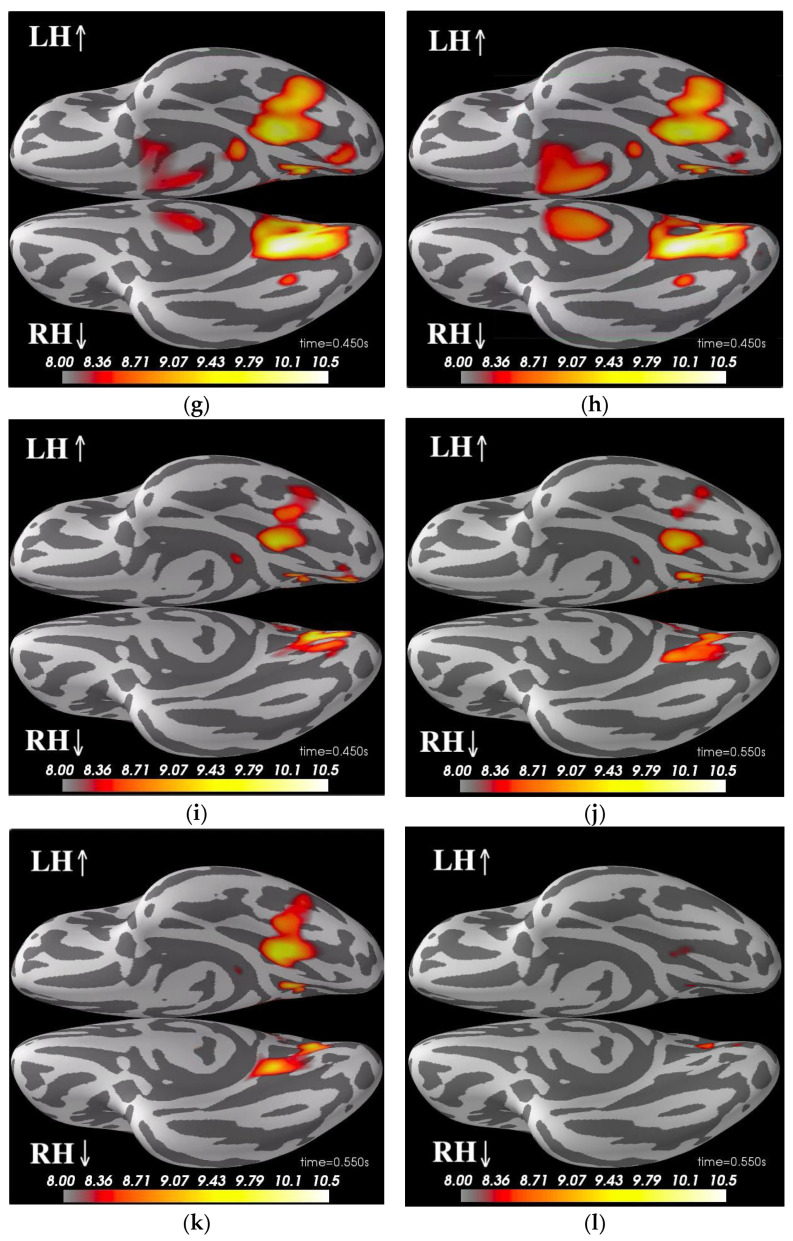
This figure shows dSPM source localization, the significant activities demonstrated here are: (**a**) famous condition IMF4 at 150 ms in left hemisphere (LH); (**b**) famous condition IMF4 at 150 ms in right hemisphere (RH); (**c**) unfamiliar condition IMF4 at 150 ms in LH; (**d**) unfamiliar condition IMF4 at 150 ms in RH; (**e**) scrambled condition IMF4 at 150 ms in LH; (**f**) scrambled condition IMF4 at 150 ms in RH; (**g**) famous condition IMF5 at 450 ms in ventral view; (**h**) unfamiliar condition IMF5 at 450 ms in ventral view; (**i**) scrambled condition IMF5 at 450 ms in ventral view; (**j**) famous condition IMF6 at 550 ms in ventral view; (**k**) unfamiliar condition IMF6 at 550 ms in ventral view; (**l**) scrambled condition IMF6 at 550 ms in ventral view.

The dSPM source localization of significant patterns before pairwise comparison for each condition is provided in Figure 3. The activities on IMF4 were mainly in the time window of 150 ms to 250 ms, while IMF5-6 involved later activities in the ventral area. Overall, other than the expected occipital and temporal cortex activation, the left hemisphere also showed a clearer pattern of parietal activities than the right hemisphere in the familiar and unfamiliar condition regardless of IMFs.

### 3.3. The Simulated Evoked Responses

**Figure 4:**
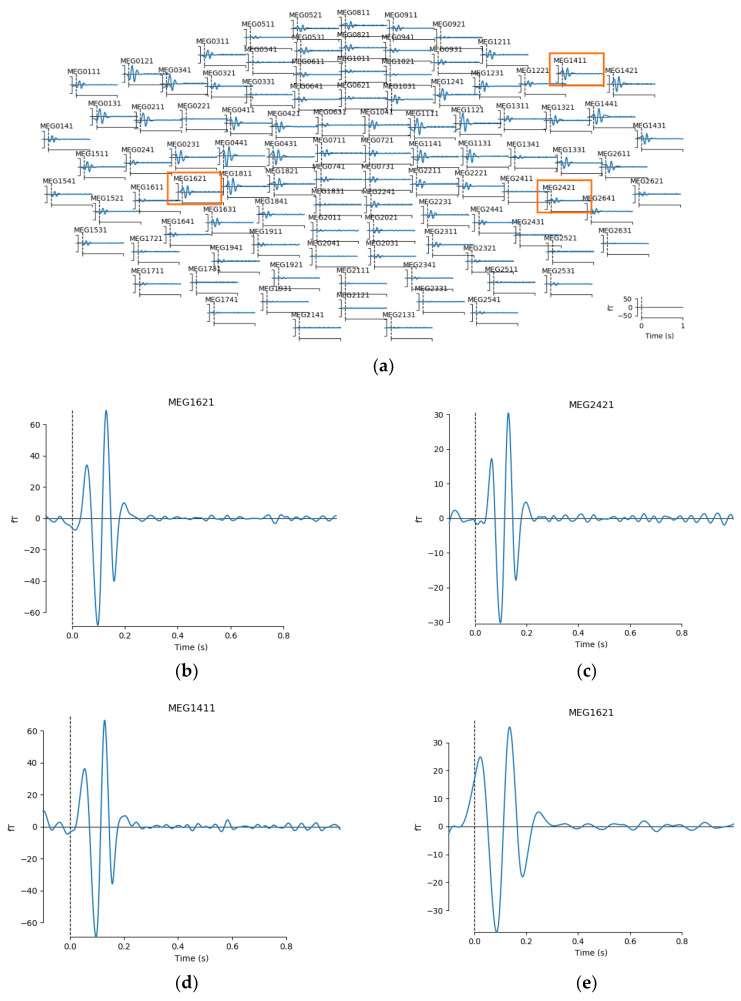

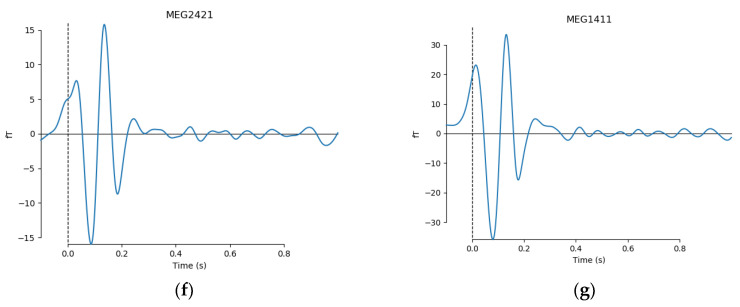
This figure shows MEG activity patterns from sensor level analysis. Here we see: (**a**) location of the selected channels; (**b**) IMF5 activities at channel 1621 (left supramarginal); (**c**) IMF5 activities at channel 2421 (right supramarginal); (**d**) IMF5 activities at channel 1411 (inferior frontal); (**e**) IMF6 activities at channel 1621; (**f**) IMF6 activities at channel 2421; (**g**) IMF5 activities at channel 1411.

**Figure 5:**
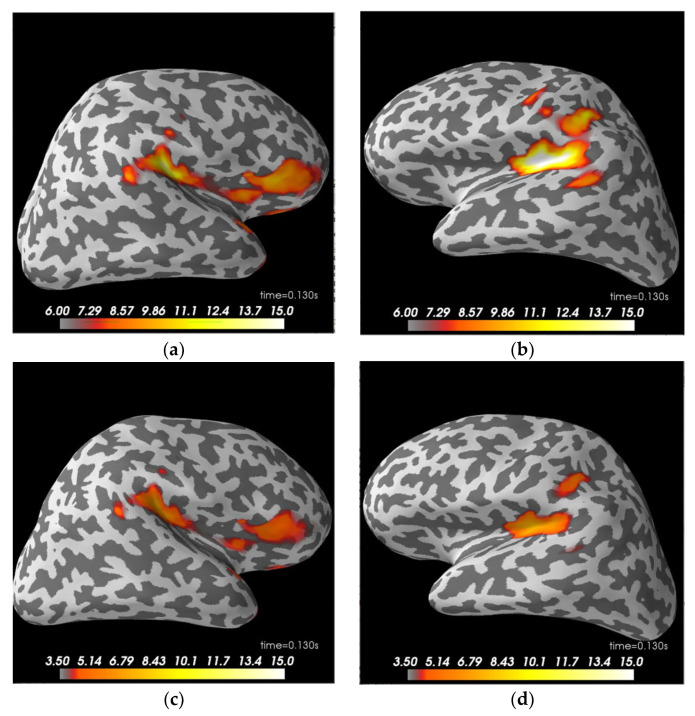
This figure shows dSPM source localization of the simulated data. IMF5 appears to capture higher level of activities than IMF6. (**a**) IMF5 at 130 ms in the RH; (**b**) IMF5 at 130 ms in the LH; (**c**) IMF6 at 130 ms in the RH; (**d**) IMF6 at 130 ms in the LH.

Next on, after processing the simulated data through our pipeline, it appeared that the simulated theta and delta activities were mainly distributed across IMF5 and IMF6 as in our actual study. Thus, our following analysis focused on these two IMF components. From sensor level analysis, we could see that the evoked response showed larger peaks at around 100–140 ms. Here we present temporal view of activities from MEG channel 1621 (left supramarginal), 2421 (right supramarginal), and 1411 (inferior frontal) for both IMF5 and IMF6 along with a sensor location map as seen in Figure 4. Source level analysis on the simulated data showed that for both IMF5 and IMF6, the main focal area of activities was in the frontal region of the right hemisphere, where we simulated the theta activities to be, while IMF5 appeared to capture more intense parietal patterns than IMF6 (Figure 5).

### 3.4. Statistical Results

**Figure 6:**
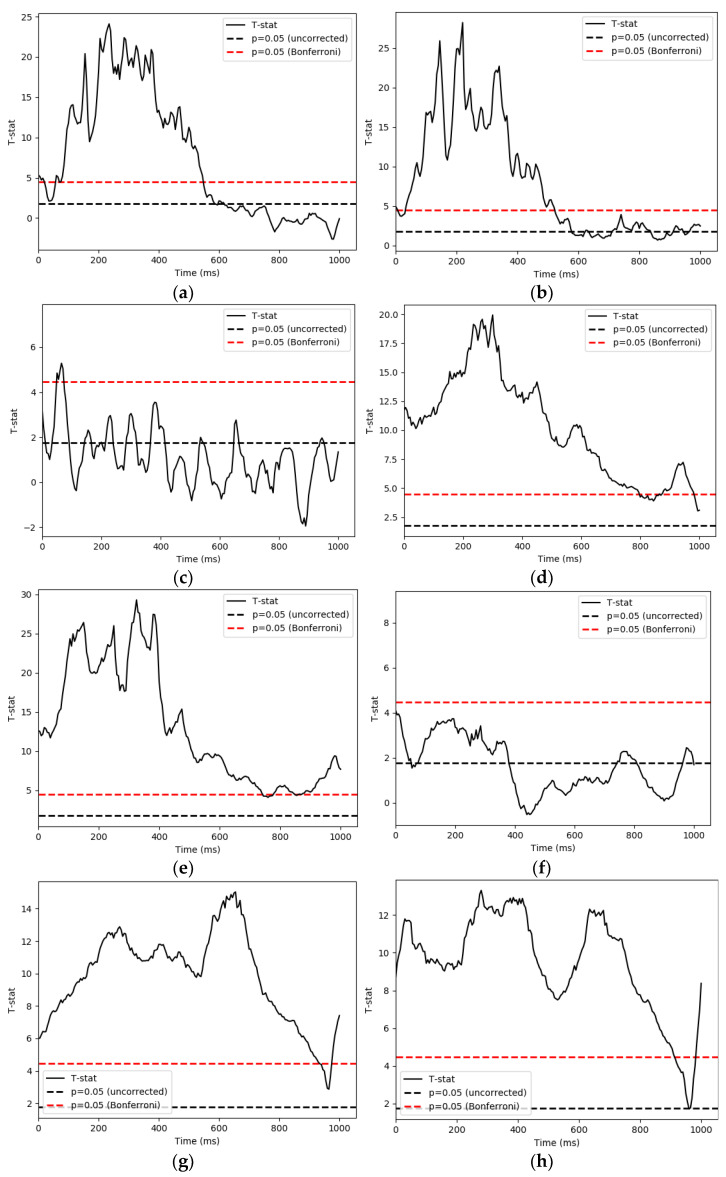

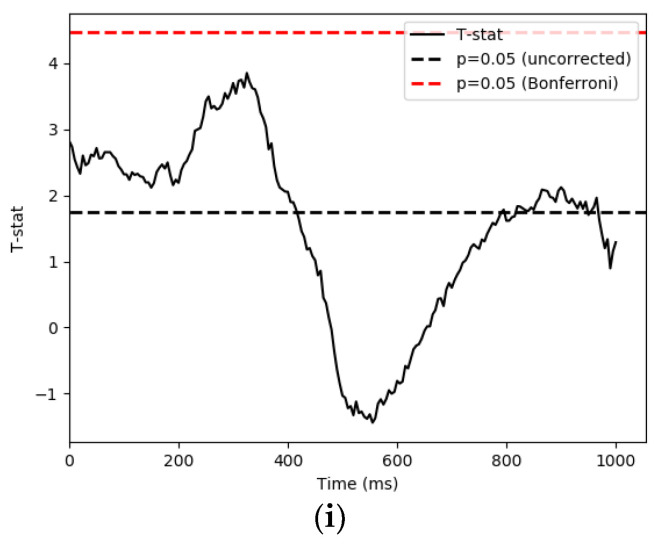
This section shows the statistical results of significant pattern differences between conditions. The *x*-axis is time, while the *y*-axis is t score. (**a**) famous and scrambled pairing (FS), IMF4; (**b**) unfamiliar and scrambled pairing (US), IMF4; (**c**) famous and unfamiliar pairing (FU), IMF4; (**d**) famous and scrambled pairing (FS), IMF5; (**e**) unfamiliar and scrambled pairing (US), IMF5; (**f**) famous and unfamiliar pairing (FU), IMF5; (**g**) famous and scrambled pairing (FS), IMF6; (**h**) unfamiliar and scrambled pairing (US), IMF6; (**i**) famous and unfamiliar pairing (FU), IMF6.

We used a parametric one sample T-test and Bonferroni correction to control the false discovery rate in retrospection. The time series of each condition and IMF’s significant t-values are demonstrated in Figure 6. We then used 1000 permutations with a t-threshold of 2 for the tests. The significant threshold was set at *p* < 0.05 (one-tailed). As the signals were downsampled from 1000 Hz to 200 Hz for analysis efficiency, we recalculated our time bins in the following reporting.

#### 3.4.1. Whole Brain Activities

Cluster-based permutation tests on the decomposed signals revealed that: (1) On IMF4, the famous and scrambled (FS) conditions showed significantly different activities from 45 ms to 685 ms (*p* = 0.001). The unfamiliar and scrambled (US) conditions differed from 0 ms to 680 ms(*p* = 0.001); and distinct activities between the famous and unfamiliar pairing (FU) were detected starting from 30 ms to 110 ms and from 140 ms to 190 ms (*p* = 0.001; *p* = 0.009). (2) On IMF5, the FS conditions showed a significant difference in patterns from 0 ms to 1105 ms (*p* = 0.001). US conditions were also revealed to have distinct activities in the same time window as the previous comparison (*p* = 0.001). As for the FU comparison, two clusters from 0 ms to 145 ms, 185 ms to 480 ms led to significant values with a *p* value below 0.05 (*p* = 0.009; *p*= 0.008). (3) Finally, for IMF6, analysis indicated that the FS conditions differed between their patterns of activation during 0 ms to 1105 ms (*p* = 0.001). The US conditions differed from 0 ms to 1060 ms (*p* = 0.001). The FU conditions were detected to differ during the time window of 50 ms to 505 ms (*p* = 0.002).

#### 3.4.2. The ROIs

We analyzed the activities occurring in the left (LH) and right hemispheres (RH), respectively, for each ROIs.

1. Fusiform

Activities on IMF4 showed pattern differences from 185 ms to 260 ms and from 270 ms to 515 ms in the LH for the FS conditions comparison (*p* = 0.005; *p* = 0.001), while a cluster was detected starting from 205 ms to 530 ms in the RH (*p* = 0.001). For the US conditions, four significant clusters were detected in the LH, spanning from 65 ms to 110 ms, 210 ms to 260 ms, 270 ms to 400 ms, and 420 ms to 490 ms, respectively (*p* = 0.029; *p* = 0.007; *p* = 0.001; *p* = 0.006), while two were detected in the RH, starting from 125 ms to 265 ms and 270 ms to 445 ms (*p* = 0.006; *p* = 0.002). For the FU conditions, distinct patterns occurred from 405 ms to 445 ms in the LH (*p* = 0.022); and no significant activity deviation was detected in the RH. IMF5 signal analysis of the FS conditions showed significant *p*-value from 195 ms to 585 ms in the LH (*p* = 0.001), and from 125 ms to 205 ms, 220 ms to 605 ms in the RH (*p* = 0.033; *p* = 0.001). The US conditions had two significant clusters in the LH, starting from 160 ms to 255 ms and 285 ms to 415 ms (*p* = 0.012; *p* = 0.003). The RH, on the other hand, appeared to differ between conditions significantly during similar timing with the LH, starting from 125 ms to 455 ms (*p* = 0.001). IMF5 did not reveal any significant difference between the FU conditions in both hemispheres. For IMF6, only the FU comparison and the LH showed a significant cluster of distinct activities, starting from 135 ms and ending at 385 ms (*p* = 0.006).

2. Inferior Temporal

Our analysis on the IMF4 signals indicated that the FS conditions had two significant activity clusters in the LH, starting from 280 ms to 335 ms, and 390 ms to 445 ms (*p* = 0.01; *p* = 0.009). As for the RH, patterns differed from 210 ms to 265 ms and from 295 ms to 380 ms, starting and ending slightly earlier than those on the left (*p* = 0.004; *p* = 0.002). The US conditions also had two clusters in the LH, with the time window spanning from 220 ms to260 ms and 285 to 335 ms (*p* = 0.003; *p* = 0.015). The RH on the other hand, demonstrated distinct activities starting at the same time but ending earlier, from 175 ms to 205 ms, 220 ms to 270 ms, and 280 ms to 335 ms (*p* = 0.048; *p* = 0.005; *p* = 0.005). Finally, the FU conditions only showed a significant activity pattern in the LH on IMF4, starting from 400 ms to 445 ms (*p* = 0.004). Moving on to IMF5, analysis revealed that for the FS comparison, the LH activities significantly differed between conditions from 200 ms to 280 ms (*p* = 0.005), while the RH differed between conditions from 265 ms to 440 ms (*p* = 0.002). The US conditions had two significant clusters in the LH and one in the RH. The significant time window occurred from 15 ms to 95 ms and 185 ms to 260 ms in the LH (*p* = 0.013; *p* = 0.027), and from 285 ms to 430 ms in the RH (*p* = 0.001). For the FU condition comparison, no significant activities were detected from the IMF5 signals. Lastly, for IMF6, the LH differed in patterns between the FS conditions from 250 ms to 390 ms (*p* = 0.021). The US 270 conditions did not show any significantly distinct activities. For FU conditions, only the LH revealed distinct patterns, starting from 165 ms and ending at 405 ms (*p* = 0.001).

3. Entorhinal

Analysis of the decomposed signal showed no significant activities with a *p*-value lower than 0.05 in both hemispheres for the FU comparison. As for the other condition pairings, IMF4 revealed pattern differences between FS during 220 ms to 255 ms in the LH (*p* = 0.004), and 220 ms to 260 ms, 355 ms to 400 ms in the RH (*p* = 0.025; *p* = 0.025). Meanwhile, the time window of significant differences between the US conditions spanned from 225 ms to 255 ms (*p* = 0.02) in the LH, and a cluster was detected in the RH, which started from 300 ms to 340 ms (*p* = 0.014). For IMF5, FS yielded activity differences from 190 ms to 280 ms in the LH (*p* = 0.009), and from 220 ms to 300 ms in the RH (*p* = 0.02). The US conditions differed in the LH from 330 ms to 390 ms (*p* = 0.007; *p* = 0.001), and from 100 ms to 180 ms in the RH (*p* = 0.043). For IMF6, no significant activity differences were detected between any two out of the three conditions.

4. Frontal

Our analysis indicated that IMF4 revealed no significant activity between the conditions. IMF5 analysis, on the other hand, indicated that the FS conditions differed in the LH, starting from 95 ms and ending at 165 ms (*p* = 0.012). The FS conditions also significantly deviated between their activity patterns on IMF6. A cluster was detected in the LH from 230 ms to 370 ms (*p* = 0.028).

5. Medial Orbital Frontal

IMF4 signals showed no significant activities below *p* = 0.05 in the FS and FU pairing conditions. As for the US conditions, analysis revealed distinct activities occurring between 220 ms to 250 ms in the LH, and 220 ms to 255 ms in the RH (LH: *p* = 0.039; RH: *p* = 0.006). For IMF5, FS yielded activity differences from 150 ms to 250 ms in the LH (*p* = 0.016), and from 205 ms to 300 ms, 920 ms to 1095 ms in the RH (*p* = 0.016; *p* = 0.002). The US conditions differed between 340 ms and 420 ms in the RH (*p* = 0.04). Moving on to IMF6, pattern differences between the FS conditions occurred from 200 ms and ended at 365 ms in the LH (*p* = 0.031).

6. Temporal Pole

Analysis on the decomposed signals showed no significant activity differences below *p* = 0.05 for the US, FU conditions on IMF4. Meanwhile, the FS conditions had a significant cluster in the RH, starting from 365 ms and ending at 400 ms (*p* = 0.033). IMF5 analysis revealed no pattern distinction above significant level between any two out of the three conditions. For IMF6, comparing FS conditions revealed activity deviation from 555 ms to 720 ms in the LH (*p* = 0.012). The activity differences in this area appeared to occur later than in the other analyzed ROIs. Lastly, we compared the significant levels of clusters in the left and right hemispheres of these ROIs.

**Figure 7:**
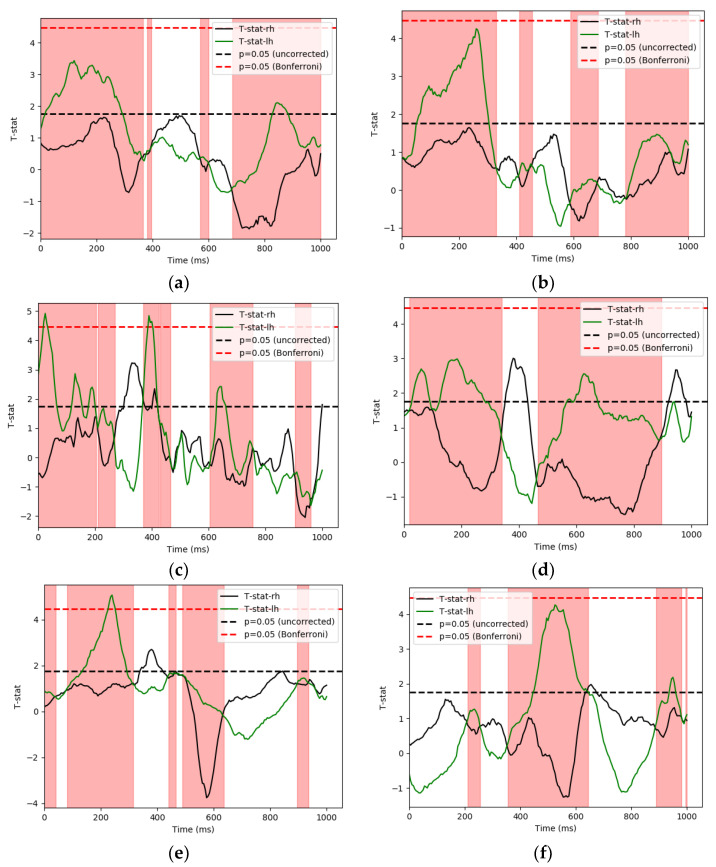
Here we compiled the activity pattern differences in the LH and RH. The *x*-axis is time, and the *y*-axis is t score. The red area is where the t-statistic level in the LH is larger than the RH. (**a**) FU pairing activity differences on IMF6 in the fusiform area; (**b**) FU pairing activity differences on IMF6 in the inferior temporal area; (**c**) FS pairing activity differences on IMF5 in the frontal area; (**d**) FS pairing activity differences on IMF6 in the frontal area; (**e**)FS pairing activity differences on IMF6 in the inferior temporal area; (**f**) FS pairing activity differences on IMF6 in the temporal pole.

As shown in Figure 7, activity differences in the LH appeared to generate scores that lead to higher classification accuracies when used to predict stimuli labels in certain areas. This effect is most evident on IMF6 involving fusiform and inferior temporal area for the FU pairing comparison, as well as the inferior temporal area, frontal and temporal pole for the FS comparison.

## 4. Discussion

When analyzing brainwave signals, it is important to consider their nonlinear and nonstationary properties in nature to minimize distortion of the oscillatory components. EMD is an adaptive approach that is capable of decomposing data with nonlinear characteristics into finite components. In this paper, we aim to provide a practical demonstration of EMD and its combinatory use with neural decoding.

We performed our analysis on open-source MEG recordings from a facial recognition experiment [5]. Although energy levels fluctuate, the corresponding frequencies at the peak of energy for each IMF were very consistent across all participants. Through HHT spectral analysis, we could not only visualize individual differences in detail, but could also identify shared properties among certain conditions or groups of participants. The method therefore further provided insight to stable features among subjects.

After decomposition, we first compared the activities between pairs of conditions (famous vs. scrambled, unfamiliar vs. scrambled, famous vs. unfamiliar), and detected significant pattern differences on IMF4, IMF5, and IMF6 for all pairings. Averaging the IMF peak energy distribution among all participants revealed that while IMF4 corresponded to the range of theta wave, IMF5 and IMF6 corresponded to that of delta. It is noteworthy that although previous studies often either had had difficulties identifying or had ignored the differences between the famous and unfamiliar face conditions [4,8,9,10,11], our data processing procedure led to significant distinction between the two. These results suggested that HHT could potentially capture features that were overlooked by other transforming methods.

Next on, in order to pinpoint the regions and specific frequencies that are involved in generating distinguishable pattern differences during the recognition process, we analyzed certain ROIs. Starting from the fusiform area, we saw that FS and US pairings had similar time windows of significance, approximately starting from 100 ms and ending at 500 ms on the same oscillation bands, IMF4 and IMF5. For FU, however, the patterns appeared to be different from the other two pairings. Only the left hemisphere showed distinction between their activity during early timings on both IMF4 and IMF6. FU pairings also showed similar patterns in the inferior temporal cortex (IT), which is an area involved in the visual pathway and visual objection recognition [18]. Importantly, it is also where the fusiform area is located. Meanwhile, the time window of significance of FS and US were shorter and ended earlier in the IT compared to what happened in the fusiform. The FS condition additionally involved brief IMF6 activities.

We then analyzed the entorhinal cortex, the gateway between neocortex and hippocampal formation. Our analysis captured significant clusters on both IMF4 and IMF5 for both the FS and US pairings prior to 200 ms, suggesting that the involvement of the memory system could happen quite early in time, irrespective of whether the stimulus’ identity was recognizable or not. Past neurophysiological studies have shown that the frontal cortex is one of the key areas that mediates face recognition memory and information integration [19,20]; therefore, we assessed the frontal area as one of our ROIs. The results showed that only the FS comparison pairing manifested significant pattern differences between their activities. The patterns emerged in the LH, within the range of delta waves (IMF5, IMF6). We were intrigued as to why the US pairing did not demonstrate similar significance, considering that both cases of facial conditions should require the involvement of a holistic integration process of facial features. As the difference be-tween famous and non-famous faces lies in participants’ familiarity to the two, one possible explanation could be that identity of famous faces is activated from memory and fed into the frontal area rapidly upon exposure.

Seeing the importance of identity recognition during the process, we analyzed the temporal pole, which is an area that consistently activates upon encounter to famous and personally familiar faces [21]. While only the FS pairings showed significant pattern difference in this area, which was similar to what we saw in the frontal cortex, the activities demonstrated in both hemispheres instead of LH only and manifested on IMF4 and IMF6 instead. This result in turn creates some open questions for the area mapping of facial memory activation. In the future, connectivity analysis between areas may aid in further understanding for this observed difference limited to the famous versus scrambled conditions. When it comes to information processing, the brain works as a complex dynamic system with temporal and spatial variability [22]. As far as the authors are concerned although there are plenty of pathological case studies that performed connectivity analysis using face and facial emotion recognition tasks, less were focused on the familiarity and identity recognition aspects of facial input for healthy participants [23,24,25]. As a result, the anatomical routes and connectivity patterns of the brain when dealing with person-related information are currently not well understood yet [23]. Using integrative and multi-subject analysis tools on EMD processed data, we could move a step forward from viewing regions of interests separately as we did in this study [14,26,27]. By doing so, the analysis could potentially provide new insights into the involvement of memory and instantaneous input from both temporal and spatial aspects during the identity recognition process.

Next on, we looked into the activities originating from the medial orbitofrontal cortex (OFC), an area involved in associating stimuli and reward [28,29]. One point to mention is that OFC was shown to be activated when people were exposed to attractive faces [30]. The experiment method in this study asked participants to judge the symmetry of faces, and symmetry is one of the criteria that humans base their judgement of overall attractiveness on [31,32]. The analysis result of medial OFC showed that for the FS and US pairing, both hemispheres showed distinguishable pattern differences, though on different oscillation bands and timing. During later timing of the FS pairing, the RH was additionally recruited from 800 ms until the end of the epoch. We suspected late feedback of person identity to attractiveness judgement takes place in the right hemisphere when viewing famous faces as opposed to scrambled faces.

In summary, the FS and US condition pairings differed mainly on theta wave (IMF4) and higher frequency delta (IMF5), while the FU comparison showed activity pattern deviation mainly on lower delta (IMF6) and a shorter time window of theta. Different from Wakeman and Henson’s original study, which did not find clusters that survived statistical testing correction for the FU conditions [5], we saw a significant time window prior to 100 ms that passed the criteria of Bonferroni correction on IMF4 in Figure 3. Through our analysis procedure, we were further able to pinpoint the area, timing, and specific frequency range that were involved with the observed significance. These results provide evidence that the utilization of nonlinear decomposition processes could bring upon more sensitivity to meaningful oscillatory components.

In the next part of our analysis, we compared the activity differences arising from the two hemispheres. Contrary to common expectation of a right hemisphere dominant pattern in differentiability [33,34,35,36], we observed either no significant hemispheric difference or left hemisphere dominance, most obviously shown in the case of FU comparison. Activities differed on IMF6 in the LH fusiform and the inferior temporal cortex as mentioned earlier. No significant pattern differences were observed on the right. Meanwhile, the FS pairings also showed similar effects on IMF5 and IMF6 in the inferior temporal cortex, temporal pole, and frontal cortex. We would like to propose two possible explanations to our analysis results above. First of all, according to a prevailing theory mentioned by Junior et al., in their review work on the topic of facial recognition, it is possible that “the right hemisphere processes faces in an integrative and comprehensive manner, whereas the left hemisphere is responsible for facial features” [37]. This would correspond with how significant pattern differences were only evident in the LH when comparing the familiar and unfamiliar conditions, which guaranteed distinguishing of facial features as both stimuli types were regular faces. Nonetheless, this theory does not explain why significant activity level differences were not observed in the unfamiliar and scrambled face pairings. This in turn led us to a second possibility: verbal label assignment of identity. Past fMRI studies have demonstrated the representation of both words and faces in the fusiform gyrus [38]. Meanwhile, the left FFA had specifically been suggested to be susceptible to perceptual learning, suggesting that it processes abstract concepts in addition to faces [39,40]. One notable difference between the famous and unfamiliar stimuli is the recognizability of facial identity. It is possible that the famous condition differed from the other two by occurrences in the brain during assignment of identities to facial images.

Although one may argue that the right hemisphere could be highly activated in all conditions, leading to difficulties in detecting relatively slight increase or decrease in activities between pairings in the RH, there is still no denying that the LH revealed meaningful distinguishing features. The left hemisphere is shown to be significantly involved in the facial recognition process quite equally in this experiment, if not more, than the right hemisphere. Past research working on face versus object recognition had also shown similar patterns of bilateral effect instead of right lateralization [41]. Nonetheless, since different methods and experimental task designs were employed in the study and our analysis, further verification and testing were needed. Future studies could explore the extent to which HHT could capture such previously unnoticed details that could go under the radar while employing linear methods on nonlinear brainwave signals.

There are limitations in our analysis. In Figure 6, we saw that after HHT processing, there was some signal distortion at the end of epochs that should be handled with caution. Additionally, the original data were preprocessed with MaxFilter 2.2 (Elekta Neu-romag) [5], therefore flattening the IMF1 energy curve for every participant as seen in Figure 2. We were thus unable to determine whether any meaningful information was carried by gamma waves during this facial recognition task. Another limitation that we encountered is the inevitable inverse problem of source reconstruction. Considering the relatively new stage of exploration on nonlinear methods, source reconstruction that are specifically customized to nonlinear input is rare, if any, as far as the authors are concerned. We currently use nonlinear operation in combination with the standard inverse method when reconstructing source, which is a common procedure in the area [42,43]. Although inverse problem could be avoided by using traditional band-pass filters, or more specifically, wavelet-based decompositions, the templates that were created by such method requires at least 3–5 signal cycles of sinusoid waves. The time window is lengthy enough for multiple events of neural activities to take place, resulting in an input of questionable temporal resolution to the covariance matrix. EMD on the other hand showed great advantage in providing precise time-course information for noise covariance. Seeing its potential, we decided to test the construction results of nonlinear processed signals by using simulation models in order to determine whether our processing pipeline is reliable enough for future practical use.

We manually created two evoked delta-band (3 Hz) responses at the left and the right supramarginal gyrus and one theta-band source (7 Hz) in the right inferior frontal gyrus and processed the signals through our analysis procedure. After decomposition, source level analysis on the simulated data showed that for both IMF5 and IMF6, the main focal area of activities was indeed located in the frontal region of the right hemisphere, which is where we simulated the theta activities to be. On the other hand, IMF5 appeared to capture higher levels of parietal patterns than IMF6, which also matches the distribution of energy as IMF5 covers signals from both theta and delta, while IMF6 captures less from theta band (Figure 5). All in all, though slight noises could be observed, the focal areas of activities were successfully reconstructed. Therefore, while we look forward to future development of nonlinear-specific reconstruction software, we are confident that the current method provides a reliable level of precision for source identification.

Our decoding analysis suggested the EMD method to be more suitable for handling brainwaves than the traditional linear techniques, which could often be even more complex mathematically. At the end, we would like to highlight that EMD holds the potential of providing new perspectives to signal extracting in the cognitive neuroscience field—whether it is decomposing useful information that was not easily extractable from noises, or replicating past experiments for further details. Our motivation for the future is to explore the practical application of EMD on neural recording data.

## 5. Conclusions

In conclusion, the main contribution of the present study was that EMD, a nonlinear decomposition method, generates reliable oscillatory components for MEG-based decoding analysis. EMD method was applied to facilitate the feature extraction from MEG. The proposed method is evaluated and applied in analyzing a publicly available dataset. Our results suggest EMD successfully decomposes signals into useful components, and poses some interesting open questions on facial identity awaiting to be further explored. Aiming to provide more insights into the topic of hemispheric lateralization in different stages of identity processing, the combinatory use of EMD processed data and connectivity analysis methods would be an interesting path for us to further explore in the future.

## Data Availability

This dataset was obtained from the OpenNeuro database. It’s accession number is ds000117 and is also made available for use under a CC0 license.
